# Comparing the Impact of an Implicit Learning Approach With Standard Care on Recovery of Mobility Following Stroke: Protocol for a Pilot Cluster Randomized Controlled Trial

**DOI:** 10.2196/14222

**Published:** 2019-11-05

**Authors:** Louise Johnson, Jane Burridge, Sara Demain, Sean Ewings

**Affiliations:** 1 Stroke Unit Royal Bournemouth and Christchurch Hospitals NHS Foundation Trust Bournemouth United Kingdom; 2 University of Southampton Southampton United Kingdom; 3 University of Plymouth Plymouth United Kingdom

**Keywords:** stroke, rehabilitation, learning, attention

## Abstract

**Background:**

Although implicit and explicit learning approaches have been well investigated in healthy populations, there is less evidence regarding the relative benefits of each approach in clinical practice. Studies in stroke typically investigate single elements of an implicit learning approach (ILA; eg, reduced quantity feedback or an external focus of attention) within controlled environments. These studies predominantly evaluate performance, with few measuring this over time (ie, learning). The relevance and transferability of current research evidence into stroke rehabilitation is therefore limited.

**Objective:**

The objective of this study was to compare the ILA with standard care in the acute phase following stroke, to generate data and insights to inform the design of a definitive trial, and to understand patient and therapist perceptions of the ILA.

**Methods:**

This is a multicenter, assessor-blind, cluster randomized controlled pilot trial with nested qualitative evaluation. Stroke units (clusters) will be randomized to either ILA (intervention) or standard care (control) arms. Therapy teams at the intervention sites will be trained in the ILA and provided with an intervention manual. Those at the control sites will have minimal input from the research team, other than for data collection. Consent will be provided at the individual participant level. Once enrolled, participants will receive rehabilitation that focuses on lower limb recovery, using the designated approach. Measures will be taken at baseline, every 2 weeks until the point of discharge from hospital, and at 3 months post stroke onset. Measures include the Fugl Meyer Assessment (motor leg subsection), modified Rivermead Mobility Index, Swedish Postural Adjustment in Stroke Scale, and achievement of mobility milestones. Fidelity of the treatment approach will be monitored using observational video analysis. Focus groups and interviews will be used to gain insight into the perceptions of trial participants and clinical teams.

**Results:**

The first site opened to recruitment in February 2019. The opening of a further 5 sites will be staggered throughout 2019. Results are expected in early 2021.

**Conclusions:**

The findings from this mixed methods pilot study will be used to inform the design of a definitive study, comparing the ILA with standard care in acute stroke rehabilitation.

**Trial Registration:**

ClinicalTrials.gov NCT03792126; https://clinicaltrials.gov/ct2/show/NCT03792126

**International Registered Report Identifier (IRRID):**

DERR1-10.2196/14222

## Introduction

### Background

Regaining the ability to stand, step, and walk is an important goal for people who have experienced a stroke and is a common focus during early rehabilitation.

The process of functional recovery post stroke is underpinned by theories of motor learning, of which there are 2 broad categories—explicit and implicit. Explicit learning occurs when someone is thinking about what to do and about how to move; it is a conscious form of learning. Implicit learning occurs through trial and error and without thinking specifically about how to move; it is a subconscious form of learning. There is, already, agreement that 2 practice conditions are particularly important when differentiating explicit from implicit learning. These are as follows: (1) the *quantity* of instructions and feedback that therapists give and (2) the *focus of attention* (FOA) derived from these instructions and feedback statements [1].

Many factors can influence the process of motor learning. However, experts consider that high quantity of information and/or promotion of an internal FOA (ie, focusing on body movements) are synonymous with an explicit learning model, and that reduced quantity of information and/or an external FOA (ie, focusing on the environment) are synonymous with an implicit learning model [2]. Bias toward one or the other form of learning can be created in a number of ways, including the way that practice is structured, how the person is instructed, and how they receive feedback.

Implicit and explicit approaches have been well investigated with healthy participants. Research has shown that tasks learnt explicitly are less robust and are less likely to be retained over time than those learnt implicitly [3]. Research in sport broadly supports the view that giving excessive verbal information during task practice reduces movement automaticity [4,5] and that reducing the frequency of feedback can enhance learning [6-8]. However, a recent systematic review highlights that such benefits may be small, and the overall quality of evidence is poor [9]. In relation to FOA, there is strong evidence that people master skills more effectively if they are prompted to focus their attention toward the environment, rather than on their body [3].

Research in stroke rehabilitation is more limited. Although studies in stroke show the relative benefits of reduced quantity feedback [10] and an external FOA [11-14], limitations of study design restrict transferability and generalizability of these findings. Most studies measure performance and have not evaluated the benefits of the given approach over time (ie, learning). In 2 studies that have compared the benefits of internal and external focus conditions on longer-term practice, no group differences were found for upper limb function (trained using a robotic device) [15] or balance (trained using a balance board device) [16].

Therefore, although implicit learning is a promising concept in stroke rehabilitation, we do not know how this approach can be effectively delivered, tailored, and evaluated in a clinical setting. Likewise, given the heterogeneity of impairments caused by stroke, there may not be a single optimal approach; it is feasible that an individualized motor learning approach is necessary to maximize recovery for an individual [17,16]. We currently lack evidence regarding who benefits most from each approach and at what time point in their recovery. Despite this, observational studies have shown rehabilitation practice to be largely explicit in nature [18-22].

### Objectives

This paper outlines the protocol for a clinical trial, which will compare an implicit learning approach (ILA) with usual care during the rehabilitation of mobility in the acute phase following stroke. The focus is on lower limb recovery, that is, sitting, sit-to-stand, transfers, stepping, and gait. The broad aims are as follows:

- To establish the feasibility of delivering an ILA during stroke rehabilitation
- To test the integrity of the study protocol (pilot)
- To generate data to inform the design of a phase III trial.

## Methods

### Study Design

This is a multicenter, assessor-blind, cluster randomized controlled pilot trial, with embedded feasibility study. It also includes a nested qualitative evaluation designed to explore the views of participants and therapists. We aim to recruit 6 Stroke units (clusters) to take part in the trial. Each unit will be randomized to deliver either the ILA or standard care. Individuals within each cluster, who meet the inclusion criteria and agree to take part, will receive all of their lower limb rehabilitation using the designated approach for the duration of their inpatient stay. The study has been approved by the Berkshire Research Ethics Committee B (18/SC/0582).

### Stage 1: Understanding and Describing Baseline

The success of this study is dependent on (1) the ability of therapy teams in the intervention sites to consistently and robustly deliver the ILA to trial participants (fidelity) and (2) there being a sufficient difference in the rehabilitation delivered to the ILA group, compared with control.

To understand current practice within each unit, we will conduct an observational study at the beginning of the trial. This will take place *before cluster randomization* and thus before clinicians have received any information or training related to the ILA. We will use nonprobability sampling to video record between 6 and 10 patient-therapist dyads (exact number to be agreed locally, depending on the size of the unit/team). Each recorded session will involve a different patient-therapist pair, but the individual patients and therapists may be recorded more than once. Therapists will be asked to continue with a routine therapy session aimed at improving sit-to-stand, stepping, transfers, or gait.

We will analyze the content of these recorded sessions using a previously validated method [21]. This will give us an indication of the likely content of standard care in each participating organization and will help us to tailor the required training (for the intervention sites). By later comparing the collective content of these recordings with those taken during the main trial, we will also get insight into the scale of the differences between the trial interventions and standard care.

### Stage 2: Cluster Randomized Controlled Trial

#### Recruitment and Randomization of Clusters

Criteria for stroke unit eligibility are a dedicated unit that (1) routinely admits patients with acute stroke and (2) has a dedicated therapy (occupational therapy and physiotherapy) service for at least 5 days per week.

Stroke units do not need to provide hyperacute care to be involved, but they must admit patients within 5 days of stroke onset. Written informed consent will be obtained by the cluster guardian (senior clinician) at each site. The cluster guardian is consenting for the *stroke unit* to take part in the trial.

The unit of randomization (cluster) is the stroke unit. The trial statistician (SE) will use a Web-based randomization system to allocate sites to control or intervention.

#### Recruitment and Consent of Individual Participants

Within each cluster, all new admissions will be screened for eligibility within 72 hours. Screening will be performed by the local stroke research nurse or therapist. They will consult other members of the multidisciplinary team, if necessary, to confirm eligibility. Those that meet the inclusion criteria will be provided with verbal and written information, which they will be given a minimum of 24 hours to consider. Those willing to participate will be asked to sign a consent form.

There may be individuals who do not meet the inclusion criteria at the beginning of their stroke unit stay but regain sufficient function to meet the criteria at a later date. We will continue to monitor potential participants and will recruit up to 14 days post stroke, if eligibility changes.

### Inclusion Criteria

The inclusion criteria are as follows:

- Clinical diagnosis of stroke, presenting with lower limb paresis
- Has rehabilitation goals relating to lower limb mobility or function
- Within 14 days of stroke onset
- Medically stable
- Able to tolerate daily therapy for a minimum of 30 min per session, sit for more than 5 seconds without support, and understand and follow single stage commands.

### Exclusion Criteria

The exclusion criteria are as follows:

- Previous stroke with residual impairments
- Other neurological diagnosis (eg, Parkinson disease, Multiple Sclerosis)
- Clinically relevant premorbid disability levels (required physical assistance of 1-2 people to transfer from bed to chair and/or unable to mobilize without physical assistance of 1-2 people).

### Intervention

For those enrolled at the intervention sites, all mobility-focused rehabilitation sessions will utilize the ILA for all rehabilitation (whether delivered by a physiotherapist, occupational therapist, or therapy assistant) that focuses on sitting, sit-to-stand, standing, stepping, transfers, and walking. The content of therapy will be based on the treatment guidelines and intervention manual, which have been developed with input from an international expert group (using Delphi methodology). As this is a clinically grounded, pragmatic trial, therapists will have freedom to tailor the specific content of each treatment session to patient need, while remaining true to the ILA. Specifically, the intervention is not prescriptive with regard to the exercises and tasks that are practiced; these are selected by the treating therapist. However, the therapist will be asked to minimize the use of instructions and feedback *during* practice and to set the task up to promote an external focus. If a task is being performed incorrectly, the therapist can either provide a further instruction or amend the task to facilitate correct performance. This approach gives the therapist autonomy to tailor the content of therapy to the individual patient, while working within the framework for implicit learning that is outlined in the intervention guidelines.

Other therapy interventions, such as upper limb rehabilitation, will be provided as per usual practice. Although the content of this additional therapy will not be monitored, the quantity of other therapy, outside of the trial interventions, will be recorded and compared between groups. Frequency of treatment will be based on the usual practice of the treating hospital. The actual number of sessions received by each participant will be recorded. Specific details relating to the ILA intervention will be shared with intervention sites once randomization has taken place.

### Control

Standard care is as per the usual working practice for the stroke unit. Standard care clusters will not have access to the trial materials (eg, treatment manual) or details about the specific elements of the intervention. They will be aware of the broad aims of the study but not the specific detail of the intervention. Although standard care has been shown to be typically explicit, we will verify this through the baseline observations for each site and the ongoing fidelity monitoring (see *Monitoring Fidelity* section). Contact with the research team will be kept to a minimum. An overview is given in Multimedia Appendix 1. The guidance for standard care is based on published observational studies describing usual practice in stroke rehabilitation [1,10,11].

### Training for Intervention Sites

For sites randomized to the intervention arm, all physiotherapists, occupational therapists, and therapy assistants will be trained in the ILA. Training is anticipated to last no more than 3 hours and will be delivered by the chief investigator (LJ) as group sessions. Training will include the following:

- Theoretical background to implicit and explicit motor learning
- Research design, methodology, and process (overview)
- Content of the ILA, including video examples and case studies to highlight application
- Opportunity for discussion and questions.

Additional training sessions will be offered if new members join the team during the recruitment phase. A manual including written, photographic, and video resources will demonstrate how to adapt standard care interventions to the ILA. Therapists will be able to refer to the manual throughout their involvement in the study. Therapists’ skill in delivering the intervention will be measured as part of the fidelity monitoring (see below).

Although the wider multidisciplinary team (eg, nurses, doctors, other Allied Health Professionals) will not be asked to change their approach with patients, those at intervention sites will be invited to attend a short educational session to raise their awareness of the trial and will be provided with written information about the study and the concepts under investigation. As these professions would not typically be analyzing movement or giving specific instructions and feedback, this level of engagement is deemed appropriate and realistic.

### Duration of Treatment

Patients will be recruited as soon as eligible, up to a maximum of 14 days post stroke onset. Trial interventions will be delivered for the duration of each participant’s inpatient stay, as deemed appropriate by the treating team. This approach is pragmatic and will ensure that the intervention can be fitted into the current care pathway, but accepting that discharge will be at different times for different patients. We will record length of stay for each participant to gain a better understanding of any variability across sites.

### Bias Protection

Outcome assessors will be blind as to the intervention group. Video recording of outcome measures will be used to achieve this, with the blind assessments being conducted by a research assistant, who is not otherwise involved in the trial (EW).

Participants will be informed that the study is investigating different approaches to providing instructions and feedback to patients during rehabilitation. They will be aware that this involves differences in the amount and the type of instructions and feedback given by therapists. However, they will be blind as to whether their stroke unit is providing control or intervention. Whether or not participants have guessed their treatment arm will be explored in the qualitative interviews.

Therapists will be involved in delivering the intervention and cannot therefore be blind. As part of their training, therapists at the intervention sites will receive information about both implicit and explicit learning. We deem this to be important to engage teams in delivering the intervention to the best of their ability. We accept that this may introduce unintended bias, but consider this risk to be small. The priority is for the intervention to be delivered consistently, and an understanding of the theoretical basis and the research hypothesis will aid this.

### Monitoring Fidelity (Adherance)

We will endeavor to record all trial treatment sessions. A small and unobtrusive video camera will be used to do this. For practicality, and to avoid observer bias, the treating therapists will be asked to set up the video camera for each session.

For each individual treating therapist (at intervention sites), the first 3 video recordings will be analyzed. We will provide each therapist with objective feedback regarding their adherence to the ILA guidelines (eg, proportion of internal to external focus instruction). In cases where adherence is low, this feedback will include practical solutions on how to improve fidelity and may involve further monitoring until adherence is achieved. For all other sessions, a random sample of videos (minimum of 1 in 6) will be selected for analysis; the sample will be stratified to ensure an equal proportion of videos from each site. Videos will be analyzed using a previously validated method [21] and will be compared for coherence with the written records of the treatment session. We will use a recognized framework to guide this process [23] through the systematic and transparent identification and appraisal of potential problems and solutions relating to fidelity.

### Measures

**Figure 1:**
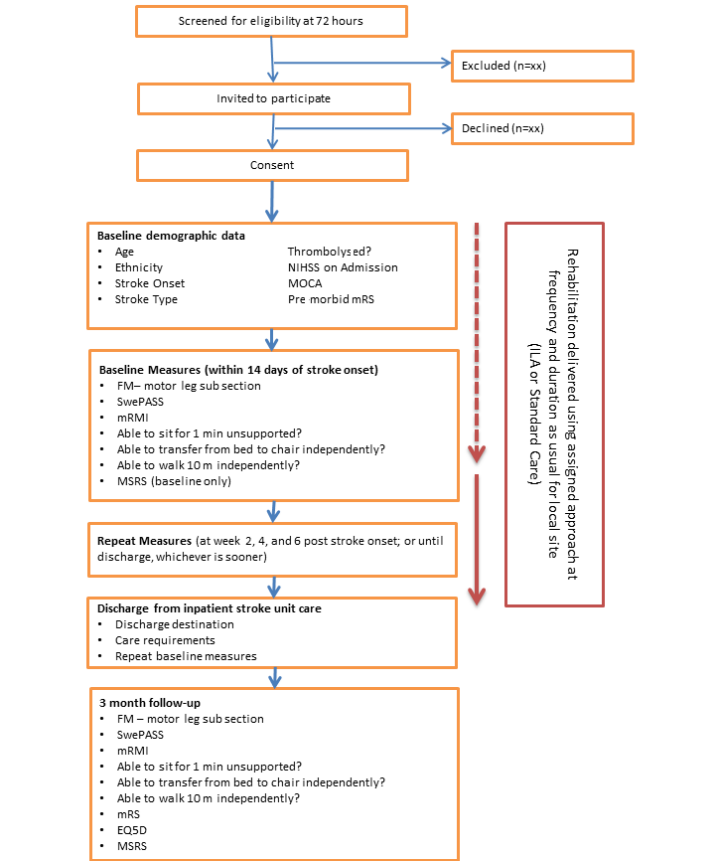
Trial flow chart. EQ5D: EuroQol 5 Dimension questionnaire; FM: Fugl Meyer; ILA: implicit learning approach; MOCA: Montreal cognitive assessment; mRS: modified Rankin Scale; mRMI: modified Rivermead Mobility Index; MSRS: Movement Specific Reinvestment Scale; SwePASS: modified Postural Adjustment in Stroke Scale; NIHSS: National Institutes of Health Stroke Scale; SU: stroke unit.

Measures will be performed and recorded by the stroke research practitioner(s) or designated clinician at each site. As the research practitioner is unlikely to remain blind to the intervention arm, all measures will be video recorded and later scored by a blinded second assessor. Frequent measures are required to understand the *rate* of change. Outcome measures have been selected with consideration of international recommendations for measurement of sensorimotor recovery in stroke [24]. Measures include the Movement Specific Reinvestment Scale [25], Fulg Meyer – motor leg subsection [26], modified Postural Adjustment in Stroke Scale (SwePASS) [27,28], modified Rivermead Mobility Index (mRMI) [29], Modified Rankin Scale [30], and the EuroQol 5 Dimension questionnaire (EQ5D) [31]. An overview is given in Figure 1.

### Proposed Sample Size

The sample size is based on estimating the recruitment rate to a desired level, while also remaining mindful of the study’s other objectives. We anticipate being able to recruit 50.0% (60/120) of all eligible people. To ensure that our estimate of this rate has a 95% confidence interval no wider than 20% (ie, ±10%), we must approach 104 people (based on exact confidence intervals [32]). As the study is cluster randomized, we must also account for this in our sample size. We aim to recruit 6 stroke units (chosen based on practicalities and ensuring we get a range of sites in which to implement the intervention, ie, hospitals of different sizes, and in different geographical locations). Assuming an intracluster correlation of 0.01, we require 120 people in total (15 per cluster). On the basis of 50% recruitment rate, we anticipate 60 people will consent to the study, allowing us to estimate retention to within ±14%. Each of the 6 sites will, therefore, be required to recruit 10 participants; keeping recruitment focused over a short time frame may also help to maintain treatment fidelity.

### Analysis

Data will be stored and managed using the Statistical Package for Social Sciences software. Analysis will be performed by the trial statistician (SE) who will be blind to group allocation.

The unit of analysis is the individual patient. As this is a pilot study, analysis will primarily be descriptive. Descriptive methods will be used to estimate practicality of factors relating to the protocol, such as recruitment (proportion of eligible people who consent to the study) and retention (completion of outcome measures at 3 months).

Fidelity of the interventions will be established by comparing the number and type of coaching statements delivered to each group. We will describe the mean number of coaching statements per person (and the breakdown of these statements as externally or internally focused) in each group. Although we expect large differences, we will not formally test the difference as the study is not designed to do so; we will instead provide an estimate of the difference with corresponding 95% confidence interval. Differences in outcome and potential effect size for the Fugl Meyer SwePASS and mRMI be calculated using confidence interval estimation.

The CONSORT (Consolidated Standards of Reporting Trials) diagram can be found in Multimedia Appendix 2.

### Stage 3: Qualitative Evaluation

To enable us to understand patient and therapist perceptions and experiences of the ILA, will we invite a subset of participants to take part in the qualitative evaluation.

#### Patient Interviews

We will invite 20 participants (10 from the intervention arm and 10 from the control arm) to take part in a semistructured interview. These will be conducted within 1 week of the final treatment session to ensure that the intervention is recent enough for the patient to recall. The purpose is to provide an understanding of patient perceptions of the ILA (compared with standard care); identify if there are any differences in the experience of those receiving the ILA, versus standard care (eg, motivation); and determine the extent to which participants were aware of what was being learnt during their treatment sessions. Interviews will be conducted by the chief investigator (LJ). They will focus on patients’ experiences of therapy and their perceptions of the benefits and disadvantages of the therapeutic style received. We will use maximum variation sampling to identify the sample and to include those with differing stroke severities (including differing levels of language and cognitive impairments), age, gender, and family/care situations.

Interviews will take place in hospital or in the patient’s home, will last for approximately 45 min, and will be audio recorded. They will later be transcribed verbatim and thematically analyzed by the chief investigator (LJ) and a second researcher. The topic guide can be found in Multimedia Appendix 3.

#### Therapist Discussion Groups

Three discussion groups (1 at each *intervention* site), involving therapists who took part in the study, will take place at the end of the trial, after all treatment sessions have been delivered. All therapists and therapy assistants who are involved in delivering the ILA will be invited to take part. The structure and analysis of these groups will be based on Normalization Process Theory (NPT) [33,34]. NPT is a sociological theory that focuses on the process by which complex interventions are made workable and integrated into everyday practice. It is concerned with identifying and understanding the ways that people make sense of the work of implementing and integrating a complex intervention (coherence); how they engage with it (cognitive participation); enact it (collective action); and appraise its effects (reflexive monitoring) [33]. The topic guide for the discussion groups will be broadly structured around the NPT framework, using a similar approach to that described previously [35]. For example, we will explore therapists’ views and beliefs relating to motor learning models, including their perceived impact and applicability within clinical practice. The insights gained from the discussion groups will give us a more valid understanding of the potential application of the ILA in clinical practice, thereby considering future implementation from the outset.

## Results

Recruitment commenced in February 2019 and will take place over an 18-month period. We anticipate results to be available in 2021. The anticipated outputs from this trial will be as follows:

- A description of current therapy practice across the 6 sites, in relation to the application of implicit and explicit learning models
- An understanding of how well therapy teams can adapt their practice and maintain fidelity of the ILA within clinical practice (by comparing the quantity and focus of instructions/feedback given between intervention and control sites, alongside quantitative data from therapist focus groups and patient interviews)
- An estimate of the difference in treatment received between the intervention and control groups, based on the frequency of instructions and feedback and their FOA
- Evidence to inform the design of a phase III trial, including the following:
  - An estimate of recruitment and retention rates to inform the future recruitment strategy
  - Agreed randomization procedures
  - Identification of appropriate primary and secondary outcome measures
  - A measure of effect size and an estimation of the required sample size
- An understanding of both patient and therapist perceptions of the ILA.

## Discussion

### Overview

Retraining of movement following stroke requires knowledge of how to apply behavioral principles of learning within the clinical setting. To date, a number of researchers have highlighted implicit learning as an important concept in stroke rehabilitation [36-40], and the factors that promote implicit learning have been defined through expert consensus [2]. Namely, implicit learning can be biased through restricting the use of instructions and feedback, adopting an external FOA, and practicing the whole task where possible [2]. Given that many people with stroke will have impairments of cognition and or language, it is feasible that ILAs, which reduce attentional demand and promote automaticity, are particularly valuable in this group. However, these concepts have not been robustly tested in clinical settings, and we lack evidence to guide rehabilitation professionals in how these approaches can be applied following stroke. We need to better understand how we can adopt fundamental principles of motor learning within the clinical setting, what works best, and for whom.

To date, much of the research in this field has investigated discrete aspects of an ILA. For example, simplifying the way in which multidisciplinary teams use instructions within an acute stroke setting [41] or promoting an external FOA [11,13,14,16]. Others have used different learning paradigms as a means of applying implicit learning in practice, including analogy learning [42,43] and errorless learning [12].

### Strengths and Limitations

Despite a clear conceptual framework for how implicit learning can be biased [40], operationalizing an implicit approach is challenging within the complexities of stroke rehabilitation. To our knowledge, this is one of the first studies to examine the implicit learning paradigm in the acute phase following stroke. It is also one of the first studies to investigate implicit learning as a complex intervention, involving multiple contributory elements. Thus, we are trialing the principle of the ILA, rather than a fixed version of it, accepting that practice may vary from therapist to therapist and from session to session. We are asking rehabilitation teams at the intervention sites to alter their whole approach to lower limb rehabilitation to maximize the bias toward implicit learning pathways and to do this throughout their intervention. The cluster randomized design is an important enabler of this and will give us the best chance of gaining fidelity among the intervention sites.

However, this approach has several methodological challenges, which will be explored through this pilot trial. In particular, how well therapy teams can maintain fidelity to an implicit approach, particularly when asked to apply it with a range of patients and over a period of time. Another key challenge relates to the “dose” of the implicit learning intervention, that is, how we ensure that there is sufficient bias toward implicit learning, such that there is a strong difference between the rehabilitation received by the intervention and the control groups in this study. This challenge is confounded by the fact that there is likely to be variability in the specific components of rehabilitation delivery received by the control groups. These differences may arise between individual therapists as well as between different sites. Our baseline period of observational data collection will allow us to understand and describe these differences, which will be further explored through the qualitative arm of the trial.

### Conclusions

Findings from this pilot study will be used to design a phase III trial. The use of qualitative and quantitative methodologies, within a study design that is embedded within clinical services, will help to ensure that the trial remains clinically grounded. This enables us to best understand if and how implicit learning models can be applied within clinical settings. It will help us to design a future pragmatic (effectiveness) trial and will maximize potential for our future findings to be readily translated into clinical practice. This phased approach aligns to the model for developing, testing, and evaluating complex interventions, as outlined in the Medical Research Council guidelines [44].

## Appendix

Multimedia Appendix 1 Overview of trial intervention.

Multimedia Appendix 2 CONSORT (Consolidated Standards of Reporting Trials) diagram.

Multimedia Appendix 3 Participant interviews - topic guide.

Multimedia Appendix 4 Peer-review report from the Department of Health, National Institute for Health Research.

## Abbreviations

CONSORT: Consolidated Standards of Reporting Trials
FOA: focus of attention
ILA: implicit learning approach
mRMI: modified Rivermead Mobility Index
NIHR: National Institute of Health Research
NPT: Normalization Process Theory
SwePASS: modified Postural Adjustment in Stroke Scale
