# Supplementary figures and images for: Comparing the Impact of an Implicit Learning Approach With Standard Care on Recovery of Mobility Following Stroke: Protocol for a Pilot Cluster Randomized Controlled Trial
Source: JMIR Res Protoc. 2019 Nov 5;8(11):e14222. doi: 10.2196/14222 (PMC6864481; doi:10.2196/14222)

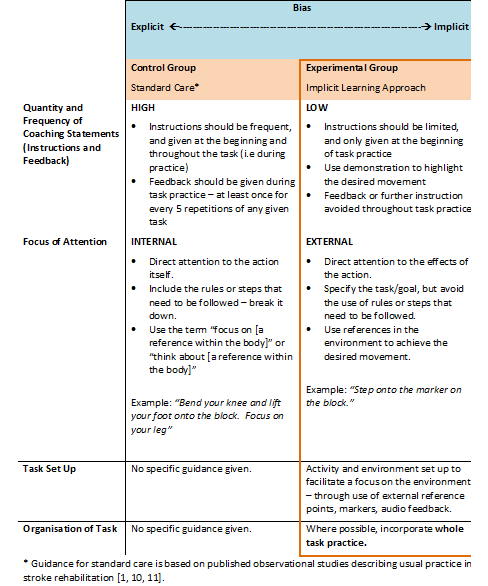

Supplement: Multimedia Appendix 1 [file resprot_v8i11e14222_app1.png]

## Appendix 2: Consort Diagram

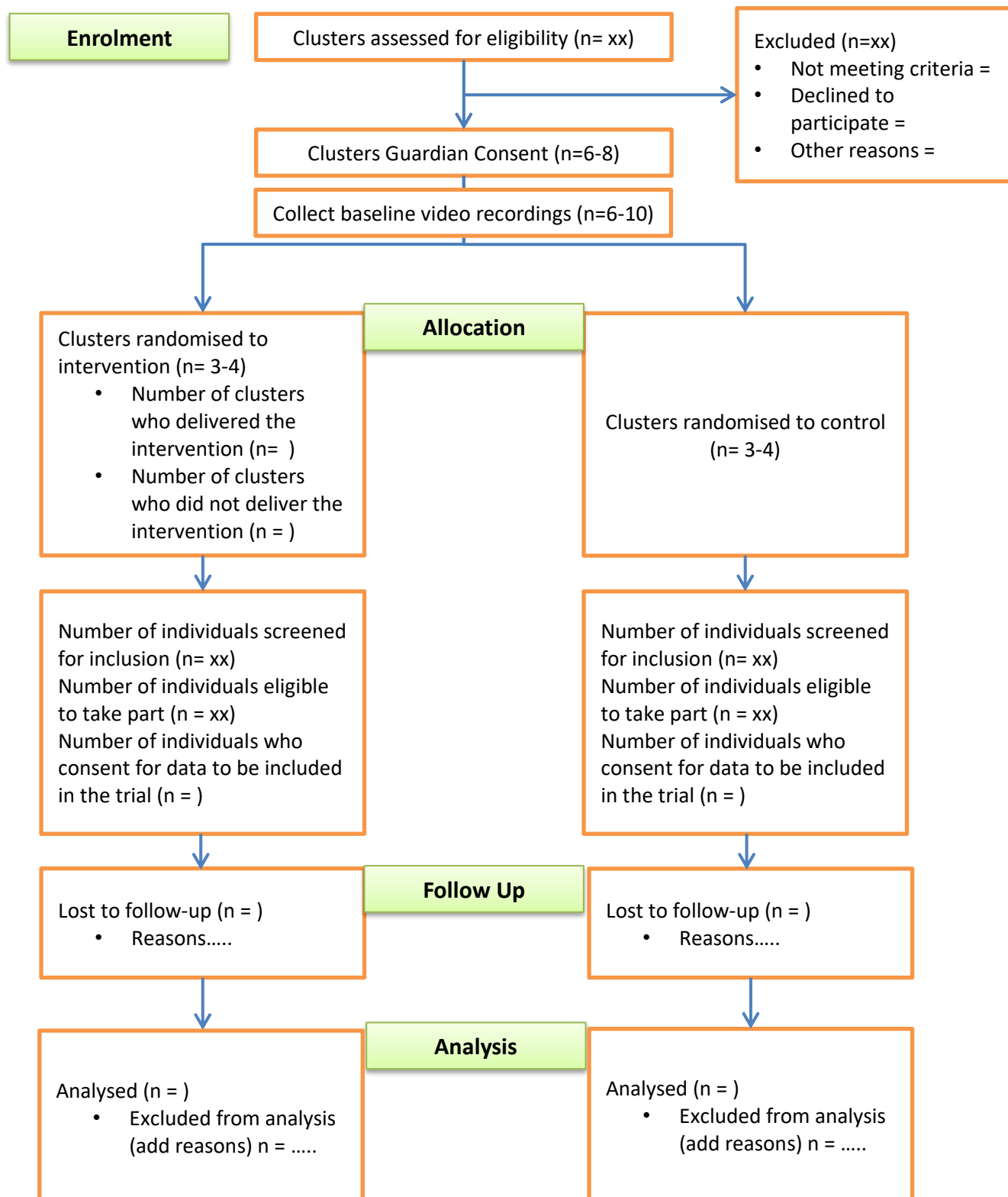

Supplement: Multimedia Appendix 2 [file resprot_v8i11e14222_app2.pdf]
