# Supplementary material for: Comparing the Impact of an Implicit Learning Approach With Standard Care on Recovery of Mobility Following Stroke: Protocol for a Pilot Cluster Randomized Controlled Trial
Source: JMIR Res Protoc. 2019 Nov 5;8(11):e14222. doi: 10.2196/14222 (PMC6864481; doi:10.2196/14222)
Supplement: Multimedia Appendix 3 [file resprot_v8i11e14222_app3.pdf]

Appendix 3: Topic Guide for Participant Interviews

**Interview Guide: Post Intervention Interviews with Patient Participants**

**Purpose:**

- To understand patient perceptions of the ILA (compared to standard care)
- To identify if there are any differences in the experience of those receiving the ILA, versus standard care (e.g. motivation)
- To determine the extent to which participants were aware of what was being learnt during their treatment sessions.

**Interview Guide:**

I'd like to ask you some questions about the rehabilitation you have been receiving whilst you have been in hospital. I am particularly interested in hearing about the therapy which has focussed on recovery of movement in your leg, and activities such as standing, stepping, walking.

| **Question** | **Prompt** |
|---|---|
| Can you tell me about what you have been doing in your physiotherapy sessions? | What have you mainly been working towards in your physiotherapy sessions? [goals]<br>Can you give some examples of the activities that you have been practising?<br>Is there anything in particular that you have been focusing on?<br>Is there anything that has been particularly difficult for you? Any types of activities or particular activities? What made it difficult? |
| You said that you have been working towards [insert activity]. When you have been practicing that, what do you think about?<br>*[understanding patient held "rules"]* | Is there anything about the movement that you focus on?<br>Do you use any other techniques to help your performance? |
| Think about how your therapist(s) worked with you during those physiotherapy sessions. How would you describe their approach? | How did you feel?<br>Is there anything that you would have liked to be different?<br>Is there anything about how they talked to you, which you felt was good?<br>Is there anything about how they talked to you, which you would have preferred to be different?<br>Did you see more than one therapist during your stay? Were they similar in their approach/or different? What did you notice that was |

| | |
|---|---|
| | similar/different? |
| Thinking about the way they gave you instructions – was it always clear what they wanted you to do? | What made it clear/not clear – can you give an example? |
| How did you know if you were doing the movement in the right way? | Did your therapist give you feedback?<br>How did they do this?  Was it helpful?  Can you give an example?<br>Would you have preferred something different/more/less? |
| Have you been practicing exercises outside of physiotherapy? | Tell me about the type of activities you have been doing…<br>How have you found this?<br>What do you think about when you are practicing outside of physiotherapy?  What do you focus on?  Is this different when the therapist isn't there?<br>How have you known if you were doing them in the right way?<br>Have you used any techniques to get feedback when the therapist isn't there? |
| How do you feel about that the progress you have made whilst here on the Stroke Unit? | What makes you feel that way? |
| What words would you use to describe your physiotherapy sessions? | If you were telling your family about your physiotherapy session, how would you describe it?<br>How have you felt during those sessions?<br>How have you felt after those sessions? |
| Is there anything else about your physiotherapy that you would like to tell me about? | |
