# Supplementary material for: Comparing the Impact of an Implicit Learning Approach With Standard Care on Recovery of Mobility Following Stroke: Protocol for a Pilot Cluster Randomized Controlled Trial
Source: JMIR Res Protoc. 2019 Nov 5;8(11):e14222. doi: 10.2196/14222 (PMC6864481; doi:10.2196/14222)
Supplement: Multimedia Appendix 4 [file resprot_v8i11e14222_app4.pdf]

NHS
National Institute for
Health Research

Summary

Reference Number	ICA-CL-2017-03-011
**Lead Applicant**	Dr Louise Johnson
**Research Title**	A pilot cluster randomised controlled trial, of an implicit learning approach (ILA) versus standard care, on recovery of mobility following stroke.

The Department of Health, National Institute for Health Research (DH NIHR) is the Data Controller under the Data Protection Act 1998 (‘the Act’). Applicants for funding should be aware that information contained in this application might be shared with other DH NIHR bodies for the purposes of statistical analysis and other DH NIHR management purposes, including targeted communications with selected groups of researchers. Applicants may be assured that DH NIHR is committed to protecting privacy and to processing all personal information in a manner that meets the requirements of the Act.

Applications are assessed using the following grading system:

5: EXCELLENT

4: VERY GOOD

3: SATISFACTORY

2: UNCOMPETITIVE

1: POOR

The complete applications (research, applicant and site/ Supervision) are graded using the following system:

Grade Criteria	Criteria	Descriptor	Outcome
A	Fully meets the criteria in all areas assessed	Excellent candidate and Application	Interview/ Shortlist
B	Meets all the assessment criteria with minor weaknesses	Very good candidate and Application	Interview/ Shortlist
C	Reasonable quality applicant however does not adequately meet the criteria	Reasonable candidate and Application	Reject
D	Not competitive	Poor candidate and application	Reject

Review Type: Panel Assessment 1

**The quality of the proposed research and its potential to have an impact on the care of the public and/or patients within five years of its completion**.
Strong

**The proposed balance of clinical work, research and teaching activities**
Strong

**The potential of the Clinical Lectureship to support the development of the applicant's skills as a clinician as well as an academic**
Strong

**Quality, scope and relevance of the review of existing evidence**
Strong

**Appropriateness and level of patient and public involvement**
Strong

**Quality of plain English summary**
Strong

**Score (1-5)**<br>**5:** EXCELLENT **4:** VERY GOOD **3:** SATISFACTORY **2:** UNCOMPETITIVE **1:** POOR
5

Comments

This was a very well structured application, demonstrating Dr Johnson used well the 0.3wte Fellowship she was awarded to develop this application. The research background is well explained and a good review of relevant evidence is summarised. Dr Johnson has undertaken a systematic review on motor learning in people with neurological conditions.

The study is a feasibility study and as such has scope to improve clinical care only if a full RCT is conducted following this trial which then demonstrates if implicit learning is more effective than explicit. As Dr Johnson states, if it does then physiotherapists will need re-training to shift from explicit to implicit learning approaches. This has wider implications for delivery of interventions by all therapists across more conditions.

The research design and method is very clearly detailed. The work packages are explained very clearly with itemised outputs from each stage. Dr Johnson has taken note of the feedback from her last application and has switched to a cluster randomised controlled trial and will train not just PTs but also OTs and therapy assistants in order to ensure patients receive a higher amount of ILA or ELA input than previously planned. The outcome measures are clearly articulated. Dr Johnson plans to monitor fidelity of the interventions, by video recording all treatment sessions with a sample of 1 in 6 will

be randomly selected for analysis, based on a previously validated method. Therapists will be given video equipment so that they can record themselves providing the intervention and forward recordings to Dr Johnson. Standard care will be defined and also monitored. As part of the training, physiotherapists will be provided guidance on what constitutes standard care.

Both the quantitative and qualitative research design, methods and analyses are explained very well within the wording limit available. PPI has been considered and integrated throughout.

The application has addressed the key concerns from last year successfully.

Dr Johnson plans for 40% research, 40% clinical, 5% formal courses and 5% other training. Dr Johnson does indicate there can be some teaching within the post – of other stroke therapists, as well as undergraduates related to the study topic. She will also have the opportunity to help supervise/ mentor an MSC and a PhD student.

**Grade**
A

Review Type: Panel Assessment 2

**The quality of the proposed research and its potential to have an impact on the care of the public and/or patients within five years of its completion**
Strong

**The proposed balance of clinical work, research and teaching activities**
Average

**The potential of the Clinical Lectureship to support the development of the applicant's skills as a clinician as well as an academic**
Average

**Quality, scope and relevance of the review of existing evidence**
Strong

**Appropriateness and level of patient and public involvement**
Strong

**Quality of plain English summary**
Strong

**Score (1-5)**<br>**5:** EXCELLENT **4:** VERY GOOD **3:** SATISFACTORY **2:** UNCOMPETITIVE **1:** POOR
4

**Comments**
Potential for impact of work is high.<br><br>Previous comments seem to have been addressed re cluster trial.<br><br>However, the issue of consent is somewhat confused - first the cluster consents, then the participant. What happens if the participant does not consent? Is this consent to randomisation or to data collection only? Will those assessing eligibility be blind to the arm of the trial - as otherwise there is potential for bias if intervention arms judge ability to "tolerate daily therapy" differently from control arms, for example.<br><br>The criteria for progression to full trial are not given. What if the pilot shows the trial to be unfeasible? Or strong evidence of harm of intervention? What will happen to phase 3 then?

**Grade**
B

Review Type: Peer Review 1

**Research Proposal**
The quality of the research:<br><br>I agree that the research needs to be grounded in the MRC framework and a cluster trial is the best apporach for this type of intervention. So I would have expected the applicant to demonstrate her insight by explaining where the study sits within the MRC framework. The project is stated to be a (Phase II) feasibility/pilot trial, yet these are different designs (see the guidance on this matter ion the RfPB website) and the applicant does not state what aspects of the trial they are piloting and/or what aspects of feasibility they are testing. The proposal would be enhanced by a clear statement of the aims; objectives and research questions to be addressed by the trial. Later the design is described as a “multicentre, assessor blind, cluster (stroke unit) randomised controlled pilot trial with an embedded feasibility study”. This is different again. There is also no mention of the mixed methods approach in the design description.<br>Surprisingly for a trial to improve mobility levels the selection criteria do not state that participants need to have limited mobility.<br><br>The recruitment rate is ambitious (~1 participant/week/ site). This is feasible if one merely considers the epidemiology for mobility problems after stroke; however it rarely happens in my experience. Recruiting 50% of eligible patients within 7 days of stroke would be very challenging; many stroke survivors are too shell-shocked by a life changing event to contemplate taking part in a trial at this stage. Recruitment is much better in the 2nd week onwards. These targets would require terrifically effective recruitment personnel who are dedicated to the project (rather than 'generic stroke search nurses). Recruitment rate, effective recruitment strategies and retention rates are aspects of feasibility which should be addressed in this study.<br>The sample size and number of sites are reasonable to establish the recruitment rate, characterise the people who are recruited and feasibility of implementing the intervention in a range of contexts.<br><br>All the stated outcomes relate to the effects of the intervention. Yet this is meant to be a feasibility trial NOT an efficacy trial. These outcomes will not tell the applicant whether it is feasible to run a Phase III trial (or not) (the supposed aim). Nor will the data collected inform the deliverables stated later in the research plan. Oh dear.<br>It is unclear why the modified Rankin and Stroke Impact Scale are being measured and why only at follow up. How do they relate to the aims/ objectives/ research questions? Why are the assessments being repeated during the training period, rather than just at the end? It appears a waste of time, effort and resources to repeat measurements without good reason. How long does the training continue? Early in the application it says 4 weeks. If so, why measure at 6 weeks?<br><br>The applicants rightly identify that ensuring that HCPs deliver the ILA as expected (fidelity) is key to its efficacy. At the beginning of the application they say that the whole MDT – OT, PTs, nurses and assistants will be involved in training to deliver the ILA. They tehn say in the detailed research plan that “All trial treatment sessions will therefore be video recorded”. Really?? Will all OT, PT and nursing treatments/ interactions be video’d? How?<br><br>The plan to develop training material for the ILA is commendable but limited. There is good evidence that to maintain fidelity with a complex interventions, clinicians needs on-going support, feedback and encouragement to guide the clinicians to implement ILA - how / what to change their practice, to build teams and co-operation, tackle barriers and work out how the intervention could/should be adapted to different context. I suggest that, as well as training material, an implementation package is

needed. One of the aims of this feasibility trial should be to investigate how fidelity can be established and maintained. Not just to measure it. See the work or Louise Connell, Elisabeth Lynch, David Clarke, Fiona Jones and Sarah Tyson for details.

The potential impact of the research on patients within 5 years:

This project addresses an important topic. As the applicant explains, rehabilitation is based on motor learning (AKA skill acquisition) theories and although these are well established in healthy populations, there has been relatively little work to test the assumption that the same processes occur, in the same way, in people when recovering from stroke. If implicit learning is more effective than an explicit learning approach then there is great potential to enhance the effectiveness of stroke rehabilitation – with consequent improved patient outcomes, plus reduced hospital and on-going health and social care costs. It is a high priority topic

The proposed balance of clinical work, research and teaching activities
- most apporriate.

The potential of the Lectureship to support the development of the applicant's skills as a clinician as well as an academic

Good

Quality, scope and relevance of the review of existing evidence:

This is thorough and concise. I would have expected some linkage with what we know is effective in stroke rehab, particularly the evidence highlighting the importance of intensity of therapy and practice. This project progresses that notion by examining the importance WHAT patients practice, rather than merely how much. However this is merely missed opportunity, rather than a fatal omission

Appropriateness and level of patient and public involvement.

Has had comprehensive input in the development of the proposal and clear plans for how this will continue throughout the project.

Quality of plain English summary

Explains a complex idea. Although some more details about what would be involved for participants would be useful. Also has muddled PPI and participation in the study.

**Grade**
B

Review Type: Peer Review 2

Research Proposal
This is a re-submission in which the PI has gone to considerable lengths to revise the application appropriately. The proposed work is important and necessary, given (1) the current hunger for new approaches to movement rehabilitation and (2) the lack of rigorous examinations of implicit approaches to stroke rehabilitation (see, for example, a recent review by Kal et al, 2016, in PlosOne). I find the rationale for the work to be strong and the approach more than feasible for a pilot trial.<br><br>I have two doubts, which I feel it important to raise, although I do not feel that they are insurmountable.<br><br>The first doubt may show my misunderstanding of the methodology to be used. To avoid contamination, a cluster randomised trial is proposed, in which 'all' patients at the four units randomly allocated to an implicit approach will be involved in the treatment. A patient may, however, opt out of such treatment, preferring instead to have usual treatment. How will this be dealt with ethically and in order to continue to avoid contamination.<br><br>Second, clinicians in the implicit training clusters will be encouraged to reduce the extent to which they provide explicit instructions during rehabilitation and encourage an external focus of attention by patients. This is easier said than done. As Masters and colleagues (e.g., Masters & Maxwell, 2008) have highlighted on many occasions, contingencies, such as pressure, injury, boredom or movement problems can cause individuals to adopt an internal focus of attention and become extremely explicit about their movements. Merely reducing the number of explicit instructions provided and encouraging an external focus is probably not sufficient to override such tendencies. Additionally, clinicians or therapists may find it difficult to suppress habitually explicit responses. Why then has the applicant not considered the use of other forms of implicit motor learning developed by Masters and colleagues, such as analogy instructions, dual-task training or even error-reduced approaches?

Grade
A

Review Type: Peer Review 3

**Research Proposal**
Physiotherapy practice and research in the UK relies heavily on untested *ad hominem* strategies and assertions that practices work based on personal experiences. It is, therefore, gratifying that this application is proposing to test an intervention in a randomised controlled trial. However, the intervention is based on practice in another field of endeavour, namely sport coaching, and it is not fully established how this translates across to a population of stroke survivors who are many decades older than sportspeople who are a highly motivated and, importantly, self-selected group of individuals. Is implicit learning in sports simply a Hawthorne effect? Nor is it clear how this proposed strategy would work with the widest group of stroke survivors, many of whom have communication and cognitive impairments as a result of their cerebrovascular disease and associated co- morbidities. Many of our physiotherapy colleagues may be using what the applicant terms 'explicit learning' simply because it is the only way to work with some stroke survivors, particularly those with neglect. Whilst I acknowledge the doctoral and PPI work that has gone into this application, this has largely been carried out in the applicant's centre. It would appear that this approach has been used in the chronic phase of stroke to a limited degree. This does introduce a degree of risk to the research, which means that this proposal needs to be of the highest quality. I would suggest, therefore, that a clinical trials unit should be involved at this stage, particularly with the fundamental mistake of assuming that an individually-randomised strategy would be sufficient in the initial application. The difficulties of trying to unpick the limitations of this pilot RCT to extend it to a definitive trial when a CTU has not been involved should not be underestimated.<br><br>I have two fundamental reservations about the research proposal.<br>The first is that the applicant has tried to increase the 'dose' of the intervention by extending the training to occupational therapists. Yet, this only extends to a three-hour training period for these two professions and their assistants, with a 'short education session' to others working in the stroke units. This does not suggest to me that the 'whole team' is delivering the intervention and barely increases the 'dose' especially as implicit learning strategies are only deployed in rehabilitation of lower limb function. Including the work that occupational therapists do towards improving mobility, whilst important for individual patients' outcomes, will barely increase the 'dose' of rehabilitation received by participants in the project. I would contend that much of the focus of occupational therapists is not the recovery of impaired movement as stated by the applicant, but the enhancement of occupational performance.<br><br>The second main reservation about this study is that the focus in the title appears to be on mobility, which I take to mean transferring, standing and walking, and yet the primary outcome measure is the Berg Balance Scale, which as the name suggests, purports to measure the individual's balance. The other main measure is one of posture, again, related, but distant to the desired outcome. At this stage in planning an intervention, and particularly with such a small cohort planned for recruitment over 20 months, I would have thought that a wider exploration of the outcomes from the intervention by measuring across the International Framework for Functioning, Disability and Health would have been more productive than focusing just on impairment. I would also contend that the application's assertion that adopting this intervention may enable people to return home earlier and thus save money for the NHS is premature as no data will be collected to confirm this.<br>The application contains numerous typographical errors (e.g. Harrogate, IK) and other errors (e.g. UKSF Conference 2010).
**Grade**
C

Interview Feedback

The research topic is very relevant for Stroke Rehabilitation. The application had addressed well the issues identified in the previous application that were of concern. The applicant has clearly put a great deal of thought into revising the application.

The candiate addressed clearly and well the key issues and queries that the Panel and reviewers had about your application both during the presentation and when questioned e.g. regarding the progression criteria to a full trial, the outcome measures being appropriate; screening for language and cognition ability; and assessing fidelity.

The candidate submitted and presented a well-constructed feasibility study and demonstrated a clear understanding of the methods to be used.

The Panel still had some concerns about the amount of ILA instruction patients would receive and advise further consideration of how this could be increased further to ensure all the team, as far as possible at sites, could use this approach. This is important in order to ensure that if ILA appears to be ineffective, it is not because patients received too little dose.

<u>PPI</u>

The PPI plan was strong. The plan was well structured and the use of levels were felt to be helpful. It was felt that this model could be used as a template going forwards. A good effort was made to co-produce elements of the research.

PPI in the dissemination and analysis of findings and infographics would strengthen the application as would consideration of PPI training.
